# About Usefulness of Kalemia Monitoring after Blunt Liver Trauma

**DOI:** 10.1155/2012/279708

**Published:** 2012-03-27

**Authors:** Francesco Meriggi, Paolo Gramigna, Paola Tramelli

**Affiliations:** Department of Surgical Sciences, IRCCS San Matteo Hospital Foundation, University of Pavia, 27100 Pavia, Italy

## Abstract

*Background*. The aim of this study is to investigate the evidence of hypokalemia as a suitable parameter for therapeutic decision making after severe blunt liver trauma. *Methods*. We reviewed the medical records of 11 patients (9 M, 2 F, mean age 32 years) admitted to San Matteo Hospital of Pavia between 2007–2009. All of them were victims of road accidents hospitalized for blunt liver injury and submitted to surgery. *Results*. Hypokalemia was observed in 7/11 (63.6%) patients during the preoperative period (mean value 2.91 mEq/L). Serum potassium concentration normalized in all patients at the 7th postoperative day only (*P* < 0.01). *Conclusions*. According to literature results, our study confirms that after blunt hepatic injury serum potassium levels may decrease significantly. Therefore, kalemia must be carefully monitored in order to establish appropriate treatment and avoid any complications.

## 1. Introduction

Trauma patients are shown to have a high incidence of admission hypokalemia, which predicts the seriousness of trauma itself. It is often associated to hyperglycemia and is subsequent to circulating epinephrine [1].

Serum potassium decrease is particularly frequent in young patients with blunt head trauma or spinal cord trauma and has a worsening prognostic implication in terms of morbidity and mortality [2].

Concerning blunt abdominal trauma, significant hypokalemia has been observed in patients with liver injury. On the contrary, no hypokalemia has been described in patients with other abdominal visceral injuries (e.g., spleen, mesentery) [3–5].

Besides elevation of serum aspartate and alanine aminotransferases (AST and ALT) [6, 7], there is evidence of a few-day-lasting hypokalemia [4, 5], despite the normal values of other serum electrolytes (Na^+^, Cl^−^), daily urinary potassium and blood acid-base balance.

The aim of the present study is to investigate the evidence of hypokalemia as a suitable parameter for therapeutic decision making after severe blunt liver trauma.

## 2. Materials and Methods

We reviewed a personal series of 11 patients (9 M, 2 F, mean age 32 years) admitted to San Matteo Hospital of Pavia between 2007–2009. All of them were victims of road accidents and hospitalized for blunt liver injury (III-V grade according to Moore organ injury scaling) [8] without any other abdominal visceral lesions associated (Table 1).

Within the first 12–24 hours from admission, they were all submitted to surgery for shock due to hemoperitoneum (9 cases) or peritonitis due to choleperitoneum (2 cases), not curable otherwise.

By perihepatic packing (8 cases) or total vascular hepatic exclusion (TVHE) with an overall length of 50 minutes (1 case) bleeding was controlled. In all cases to complete haemostasis and to stop bile leakage hepatic parenchymal or vascular lacerations were sutured. One patient underwent hepatic resection of segment II; in two patients cholecystectomy was mandatory.

The pre- and postoperative serum potassium levels of each patient were examined.

## 3. Results

The postoperative course was uneventful in most cases; four patients (36.4%) had respiratory complications (pleural effusion).

All patients recovered and were discharged after an average hospitalization of 12 days.

With reference to pre- and postoperative serum potassium levels, hypokalemia was observed in 7 out of 11 (63.6%) patients during the preoperative period (mean value 2.91 mEq/L). Despite the proper serum electrolytes replacement (Na^+^, K^+^, Cl^−^, Ca^++^) and the appropriate correction of the blood acid-base balance, hypokalemia was still observed in 8/11 (72.7%) patients at the 1st postoperative day (mean value 3.22 mEq/L) and in 3/11 (27.3%) patients at the 3rd postoperative day (mean value 3.3 mEq/L). For all patients potassium levels returned to normal (3.5–5.3 mEq/L) only at the 7th postoperative day (Table 1). Moreover, the standard deviation values seem to indicate a convergent path to kalemia normalization after 1 week (Table 2).

Table 2 reports the sample statistics of kalemia at different frequencies for 11 patients affected by severe blunt liver injury.

Before surgical treatment the average level of serum potassium (3.36 mEq/L) is below the minimum threshold (3.5–5.3 mEq/L). Even if the mean value of kalemia (3.88 mEq/L) is normal after the 3rd operative day, the minimal value is still below the normal threshold. Moreover, after a week, kalemia variability (4.8–3.7 mEq/L = 1.1 mEq/L) among patients is drastically reduced to half of the corresponding variability (4.3–2.1 mEq/L = 2.2 mEq/L) before surgical treatment.

This suggests convergence of kalemia to normal values already after three days for the majority of patients. After seven days, all patients have normal levels of serum potassium.

These preliminary results require a formal investigation in order to assess their statistical significance. With this purpose, tests on the difference between the sample mean of kalemia are then implemented to evaluate if the convergence to normal values, respectively, after one, three, and seven days, is statistically significant.

To realize this purpose, *T*-test and a nonparametric test, the Wilcoxon signed rank test [9], that relaxes the Gaussianity hypothesis of the observations, have been computed. The results are reported in Table 3.

Both tests strongly support the hypothesis that the level of potassium converges to the normal values after a week from surgery (*P* values are close to 0.01 in both cases). Moreover, there is a significant improvement already after 3 days (*P* values are close to 0.05 in both cases), while kalemia does not increase significantly, on average, after one day, remaining below the minimum threshold of the normality region.

Tests have been repeated excluding patients with the minimum (say 2.1 mEq/L) and the maximum (say 4.3 mEq/L) levels of potassium before surgical treatment. The results, in terms of tests significance, are close to those obtained with the whole sample.

## 4. Discussion

Liver is a particularly vulnerable organ because of its size, the fixed position in the right hypochondrium, and the strict anatomical relationships with duodenum and pancreas [10].

Patients must be carefully monitored in the intensive care unit, well equipped for rapid laboratory, radiologic (ultrasound, computed tomography, arteriography) and laparoscopic investigations [11] in order to diagnose sudden risky complications, needing a prompt treatment [10, 12–14].

Emergency surgery is contemplate in case of complications, such as shock due to hemoperitoneum and peritonitis due to choleperitoneum or to other associated abdominal visceral lesions [15, 16].

After trauma implying hepatic parenchymal injury, the consequent cytolysis should cause hyperkalemia. On the contrary, as reported in literature [4, 5], hypokalemia is observed. This evidence could not be ascribed neither to a reduced intake nor to an abnormal potassium depletion. It was rather assumed the hypothesis of an enhanced potassium transfer into the intracellular compartment, particularly into the hepatic one. Indeed, liver is provided with rich intraparenchymal adrenergic endings. In case of hepatic trauma, the stimulation of intrahepatic adrenergic fibres would cause (nor)epinephrine release with consequent *β*_2_-adrenergic receptors stimulation. This would lead to an increased cellular potassium uptake from the liver (and muscles) through a direct [17] and an indirect mechanism (hepatic glycogenolysis → hyperglycemia → insulin hyperincretion → increased cellular uptake of glucose and potassium by way of membrane-bound Na/K ATPase stimulation) [1, 2, 5, 18, 19].

The persistence of hypokalemia even after 24–48 hours from hepatic trauma could be related to a temporary secondary hyperaldosteronism [5] and to a slow reduction of (nor)epinephrine levels [1].

With reference to eleven patients belonging to our personal series (Table 1), suffering from blunt liver trauma and being submitted to surgery because of complications due to parenchymal injury, in the preoperative period hypokalemia was severe (mean value 2.91 mEq/L) in seven cases (63.6%). Despite the adequate serum electrolyte replacement (Na^+^, K^+^, Cl^−^, Ca^++^) and the proper correction of the blood acid-base balance, hypokalemia was still moderate in eight patients (72.7%) on the 1st postoperative day (mean value 3.22 mEq/L) and in three patients on the 3rd postoperative day (mean value 3.3 mEq/L). Only on the 7th postoperative day serum potassium level had completely normalized in all patients. Pre- and postoperative kalemia variations (already after 3 days and after a week from surgery) were statistically significant, with a *P* value close to 0.05 and 0.01, respectively.

According to literature results, our observations, although preliminary and numerically limited, seem to confirm that, after a severe blunt liver trauma, a reduction of serum potassium levels occurs as a plausible expression of the parenchymal damage seriousness. Moreover, a serum potassium level increase is observed after seven days from surgery. It is statistically significant and is not spuriously induced by the presence of outliers.

This evidence should encourage further clinical research in this field in order to increase the sample size and to obtain definitive results.

Finally, in case of surgical treatment, in contrast with some other extra-abdominal trauma (blunt head trauma, spinal cord trauma) [1, 2], hypokalemia, if adequately handled, does not play any worsening prognostic implication in terms of morbidity and mortality.

## 5. Conclusions

It remains of primary importance to remember that blunt liver injury can cause a statistically significant hypokalemia, even severe, whose normalization, despite appropriate treatment, could need up to one week.

Apart from its etiology hypokalemia is risky by itself. Therefore, it is important to monitor kalemia in patients with blunt liver trauma in order to prevent serious cardiac complications (arrhythmia, heart failure) [4, 20]. This recommendation seems to be useful not only in case of a surgical approach, but also when, according to clinical (hemodynamic stability; absence of peritonitis) and radiological data, a nonoperative treatment is established [10, 12, 13, 21].

## Figures and Tables

**Table 1:** Clinical series of 11 patients with blunt liver injury submitted to surgery. D1, D3 and D7 are, respectively, the 1st, 3rd and 7th postoperative day.

Patient (no.)	Sex	Age (yrs)	Preoperative kalemia (mEq/L)	D1 kalemia (mEq/L)	D3 kalemia (mEq/L)	D7 kalemia (mEq/L)	Liver injury moore grading		
1	M	38	4.3	3.3	4	4.2	IV		
2	M	29	2.8	3.2	4.3	4.5	IV		
3	M	42	2.8	3	4.5	4.8	V	Cholecystectomy TVHE	Right hepatic vein laceration
4	M	32	4	4.2	4.2	3.7	III		
5	M	21	4.3	3.4	3.4	3.8	V		Choleperitoneum
6	M	14	3.4	3.4	3.1	3.8	III		
7	F	31	3.2	3.3	3.9	4.2	IV	Cholecystectomy	Choleperitoneum
8	M	64	3.2	3.4	3.7	4.7	IV		
9	F	33	2.9	4.2	3.4	4.7	IV	Segmentectomy II	
10	M	22	4	4.2	4.2	4.3	III		
11	M	26	2.1	2.8	4	4.5	V		

**Table 2:** The sample statistics of kalemia are reported at different frequencies: before surgical treatment (Preop); the day after surgery (D1); three days after surgery (D3); one week after surgery (D7).

Variable	Mean	Std. Dev.	Min	Max
Preop	3.363636	.7117201	2.1	4.3
D1	3.490909	.4908248	2.8	4.2
D3	3.881818	.4354726	3.1	4.5
D7	4.290909	.3910359	3.7	4.8

**Table:** (a) *T* test.

Variable	$\overset{̅}{\Delta }$	Std. Err.	*T*-test	*H*_1_ : *E*(Δ) < 0
D1	−.1272	.1940	−0.656	0.2633
D3	−.5181	.2672	−1.939	0.0406
D7	−.9272	.3057	−3.033	0.0063

**Table:** (b) Rank test.

Variable	Σ*R*	Std. Dev.	Rank test	*H*_1_ : *Q*_Preop_^50^ < *Q*_*D*_*i*__^50^
D1	46	11.2250	−1.203	0.1146
D3	51	11.2308	−1.603	0.0545
D7	58	11.2472	−2.223	0.0131
